# Metastatic Osteosarcoma: A Review of Current Issues in Systemic Treatment

**DOI:** 10.1080/13577149778191

**Published:** 1997-12

**Authors:** Vivien Bramwell

**Affiliations:** London Regional Cancer Centre University of Western Ontario 790 Commissioners Road, East Ontario London N6A 4L6 Canada

## Abstract

*Purpose*. Original articles and abstracts published between January 1991 and January 1997 were selected according to specified criteria and reviewed to provide answers to five interesting questions about the systemic treatment of metastatic osteosarcoma.

*Results*.

(1) In patients with metastatic disease at presentation, what is the outcome after intensive multi-agent chemotherapy?

Historically, survival has been poor, but may be improving with the use of ifosfamide-containing regimens.

(2) Can response to new agents be evaluated better in patients who have received no previous chemotherapy?

Based on limited data, this is probably true.

(3) Is the response to neo-adjuvant chemotherapy, as determined by histopathology, similar for the primary tumor and synchronous pulmonary metastases?

With intensive multi-agent chemotherapy, good histological response rates are in the range 70–90% for both groups.

(4) What is the outcome, after intensive combined modality treatment with chemotherapy and surgery, in patients relapsing with metastases after previous adjuvant chemotherapy, and what are the important prognostic factors?

Outcome is highly variable, but 5-year survival ranges between 25 and 50% and a good outcome is more likely if recurrent disease is limited to resectable lung metastases.

(5) Can a biological agent (L-MTP-PE) prolong the time to relapse in patients with resected metastatic osteosarcoma?

Preliminary data suggest that this is possible, but more studies are required.


*Sarcoma* (1997) 1, 123–130

CARFAX

ORIGINAL ARTICLE

### Metastatic osteosarcoma: a review of current issues in systemic treatment

VIVIEN BRAMWELL

*London Regional Cancer Centre and University of Western Ontario, London, Canada*

**Abstract**

*Purpose.* Original articles and abstracts published between January 1991 and January 1997 were selected according to specified criteria and reviewed to provide answers to five interesting questions about the systemic treatment of metastatic osteosarcoma.

*Results.*

(1) In patients with metastatic disease at presentation, what is the outcome after intensive multi-agent chemotherapy?

Historically, survival has been poor, but may be improving with the use of ifosfamide-containing regimens.

(2) Can response to new agents be evaluated better in patients who have received no previous chemotherapy?

Based on limited data, this is probably true.

(3) Is the response to neo-adjuvant chemotherapy, as determined by histopathology, similar for the primary tumor and synchronous pulmonary metastases?

With intensive multi-agent chemotherapy, good histological response rates are in the range 70–90% for both groups.

(4) What is the outcome, after intensive combined modality treatment with chemotherapy and surgery, in patients relapsing with metastases after previous adjuvant chemotherapy, and what are the important prognostic factors?

Outcome is highly variable, but 5-year survival ranges between 25 and 50% and a good outcome is more likely if recurrent disease is limited to resectable lung metastases.

(5) Can a biological agent (L-MTP-PE) prolong the time to relapse in patients with resected metastatic osteosarcoma?

Preliminary data suggest that this is possible, but more studies are required.

**Key words**: *metastatic osteosarcoma, treatment, chemotherapy, surgery.*

### Introduction

With modern intensive multi-agent chemotherapy, 5-year overall survival (OS) figures for non-metastatic extremity osteosarcoma of children and young adults have improved and are in the range 40–80%.[1] Unfortunately, between 15 and 20% of patients with osteosarcoma will present with clinically detectable metastases, a proportion that has increased with sophisticated methods of detection such as computed tomography (CT) or magnetic resonance imaging (MRI). As determined by measurable clinical response, chemotherapy so far has produced rather unimpressive results in metastatic osteosarcoma.[2] Early data showed overall response rates for single agents such as doxorubicin (DOX), cisplatinum (DDP), high-dose methotrexate (HDMTX) and ifosfamide (IFOS) of between 15 and 25%, with even lower response rates, 8–15%, for agents such as bleomycin, cyclophosphamide, actinomycin D (BCD), dacarbazine, melphalan and mitomycin C.

The serendipitous discovery that high rates of necrosis could occur in tumors of patients receiving neo-adjuvant chemotherapy while awaiting manufacture of prostheses,[3] and the establishment of a clear link between a good pathological response (> 90% necrosis) in the primary and a good outcome, have significantly changed the approach to the management of osteosarcoma. Although the most dramatic results have been achieved in non-metastatic cases, some promising advances in metastatic disease (regimens incorporating IFOS,

Correspondence to: V. Bramwell, London Regional Cancer Centre, 790 Commissioners Road, East, London, Ontario, Canada N6A 4L6. Tel: + 1 519 685 8640; Fax: + 1 519 685 8624; E-mail: vbramwell@lrcc.on.ca.

1357-714X/97/030123-08 $9.00 © 1997 Carfax Publishing Ltd biological therapy) that have provided interesting strategies for early stage disease are reviewed here.

### Methods

The scope of this review is limited to articles published between January 1991 and January 1997. Previous reviews in 1987[2] and 1995[1] by the same author covered much of the earlier literature.

### *Data identification*

A computerized search of Medline and Cancerlit databases for the years January 1991–January 1997 was performed specifying the key words 'osteosarcoma' and 'chemotherapy', and limiting the search to the English language. This was supplemented by papers in the author's files and scrutiny of reference lists of previous reviews and key articles.

### *Data selection*

Publications were selected for review if they met the following criteria:

(1) patients had histologically confirmed osteosarcoma;
(2) metastases were present at diagnosis, or developed subsequently;
(3) patients received some form of systemic treatment;
(4) minimum of 11 evaluable patients were available for analysis;
(5) if survival was a principal outcome, minimum follow-up of 12 months or median of $\geqslant 24$ months.

Review articles were excluded. Reports of single institutional experience with multiple chemotherapy regimens were excluded, unless these focused on prognostic factor analyses. If several papers contained similar data the most recent and/or most informative paper was included. The reviewed studies have been organized around a series of questions for which the results have relevance. However, this grouping may not always reflect the primary purpose of the study.

In the studies selected, the majority of patients were children/young adults with common, high-grade osteosarcoma. Data on histological response in text and tables are referred to as 'good' and 'poor' corresponding to $>90\%$ necrosis and $\leqslant 90\%$ necrosis, respectively.

### Results and discussion

(1) In patients with metastatic disease at presentation, what is the outcome after intensive multi-agent chemotherapy?

Five studies are detailed in Table 1. The two largest studies with the longest follow-up[4,5] show that the outlook is poor: 18% OS at a median follow-up of 84 months and 11% OS at a minimum follow-up of 60 months. In the study from Memphis[6] the Kaplan–Meier curve indicates an OS of approximately 30% at 60 months but this study only included patients with pulmonary metastases. All of these patients had persistent disease and many would have eventually succumbed. Similarly, in the first Bologna study reporting on patients treated between 1986 and 1990[7] the disease-free survival (DFS) figure at 30 months of 26% suggests a poor long-term prognosis. The presence of bone metastases at presentation was an adverse prognostic factor[4,5] as all patients in both studies died. In a multivariate analysis, Meyers *et al.*[5] found that a better outcome was also associated with age over 21 years, response to pre-operative chemotherapy and completeness of resection at all sites of tumor. Survival was not affected by use of pre-operative chemotherapy vs immediate surgery, and did not correlate with levels of lactic dehydrogenase, alkaline phosphatase or the site of primary tumor.

Two more recent studies have produced relatively encouraging results, although follow-up is shorter. The 5-year relapse-free survival (RFS) figure of 42% (Table 2) for a series of 33 patients treated on a Pediatric Oncology Group (POG) 'therapeutic window' study (see next section) is promising.[8,9] Similarly, in a second study[10] of 23 patients treated in Bologna between 1993 and 1995 (Table 1) the projected 2-year survival was 45% (mean follow-up 24 months) and 10 patients were continuously disease-free at the time of reporting. Both these studies incorporated IFOS into intensive multi-agent regimens of HDMTX/DDP/DOX.

(2) Can response to new agents be evaluated better in patients who have received no previous chemotherapy?

Evaluating new agents in patients with poor prognosis (usually due to the presence of metastatic disease at presentation) by giving two courses of a new agent before surgery, to be followed by intensive multi-agent chemotherapy, has been referred to as 'therapeutic window' treatment (Table 2). The postulate that the activity of new drugs can better be evaluated in tumors not previously exposed to chemotherapy seems to be confirmed by the results of a POG study reported by Harris *et al.*[9] For single-agent IFOS, a response rate of 27% was observed in 33 patients treated by this method, in contrast with 10% in a non-randomized group of 30 patients relapsing after intensive multi-agent chemotherapy ($p = 0.04$). In a second study of carboplatin (CARBO), Ferguson *et al.*[11] using a mixture of clinical, radiological and pathological response criteria showed that only 1/34 (3%) patients had a partial response when all sites of disease were con-

**Table 1.** *Primary and metastases at presentation (intensive chemotherapy)*

| Institution/Group (reference) | Chemotherapy | No. patients evaluable | No. local treatment | Good response No. (%) | Outcome |
|---|---|---|---|---|---|
| SFOP, France[4] | Multiple: + DDP<br>− DDP | 48<br>25 | 60 | 21/41 (51) | 18% alive,<br>median follow-up 84 months |
| Memorial, New York[5] | HDMTX/DOX<br>BCD ± DDP | 62 | 56 | 16/35 (46) | 11% alive,<br>median OS 20 months,<br>minimum follow-up 60 months |
| St Jude, Memphis[6] | (1) Single/two drug<br>(2) Standard multiple<br>(3) Intensive multiple | 4<br>9<br>18 | —<br>—<br>— | —<br>—<br>— | median OS 4.5 months<br>median OS 12 months<br>median OS 36 months |
| Rizzoli, Bologna[7] | HDMTX/DDP/DOX | 23 | 23 | 6/23 (26) | 30 month DFS 26%<br>30 month OS 39% |
| Rizzoli, Bologna[10] | HDMTX/DDP/DOX/IFOS | 23 | 23 | 15/21 (71) | 2 year OS 45%,<br>mean follow-up 24 months |

**Table 2.** *Primary and metastases—therapeutic window* and phase II studies*

| Institution/Group (reference) | Chemotherapy | No. patients evaluable | Prior treatment | No. responders | Further treatment | Outcome |
|---|---|---|---|---|---|---|
| POG[8,9] | IFOS 2.4 g $m^{-2}$ $day^{-1} \times 5$ q 21 days | New 33*<br>Relapse 30 | None<br>Multiple | 9 (27%)<br>3 (10%) | HDMTX/DOX DDP/IFOS | 5-year RFS 45% OS 52% |
| POG[11] | CARBO 1000 mg $m^{-2}$ q 21 days | 34* | None | 8 (24%) | Surgery HDMTX/DOX DDP/IFOS | 4 alive/well<br>8 alive/disease<br>18 dead |
| Hamberg[12] | CARBO 300 mg $m^{-2}$ $day^{-1} \times 2$ q 21 days | 20 | Multiple drugs including DDP | 0 | VP 16, anthracycline | — |
| MD Anderson, Texas[13] | Paclitaxel 175 mg $m^{-2}$ q 21 days | 15 | HDMTX/DOX/ DDP/IFOS | 0 | — | — |
| Saudi Arabia[14] | VP16 50 mg $m^{-2}$ $day^{-1}$ oral $\times$ 21 days q 28 | 14 | DOX/DDP | 0 | — | — |
| SFOP[16] | IFOS 3 g $m^{-2}$ $day^{-1} \times 4$<br>VP16 75 mg $m^{-2}$ $day^{-1} \times 4$ | 25 | Multiple 18 pts prior IFOS | 3CR + 9PR (48%) | — | — |

CR = complete response.

PR = partial response.

sidered, but 8/34 patients showed a response at one or more individual sites. This was felt to be lower than might be expected for DDP.

In contrast, several groups have shown low or negligible response rates when evaluating single agents in patients relapsing after multi-agent chemotherapy. As shown in Table 2, CARBO was inactive,[12] as was paclitaxel[13] and oral etoposide.[14] Pappo *et al.*[15] retrospectively evaluated the response to salvage chemotherapy in 86 patients with recurrent or metastatic disease after at least one treatment regimen comprising chemotherapy and/or surgery. One to six post-relapse single- or multi-agent treatments were delivered. Responses were not seen from regimen 3 onwards and response rates were 13% and 7% for regimens 1 and 2, respectively. Responses were seen only with HDMTX (2/25 patients), DDP (3/18 patients), IFOS (8/20 patients) and etoposide (VP-16)/CARBO (1/1 patient).

The French Society of Pediatric Oncology (SFOP) achieved better results, with a 48% clinical response rate (2 good pathological responses) in 25 patients receiving combination chemotherapy (IFOS/VP-16) at relapse after adjuvant HDMTX/DOX.[16]

(3) Is the response to neo-adjuvant chemotherapy, as determined by histopathology, similar for the primary tumor and synchronous pulmonary metastases?

Biagini *et al.*[7] were able to compare histological response between the primary tumor and metastases in all 23 patients in the first Bologna study. Overall, 71 metastatic nodules were removed and a good response was observed in 17 (24%) of these. In six patients showing a good response in the primary tumor, the response was also good in 12/21 (57%) metastases. Conversely, in 17 patients showing poor response in the primary only 5/50 (10%) metastases showed a good response. The rate of good response in the primary tumor was lower (26%) in this series of patients presenting with metastatic disease than in 168 patients with localized disease receiving similar chemotherapy (76%).

However, in a second Bologna study,[10] a good response was observed in a higher proportion of metastatic lesions, 71/79 (89%). This was also true for the primary tumors with 11/15 (73%) responding. Although there was no significant difference in the proportion of good responses between metastatic and primary tumors, the incidence of complete necrosis was significantly better ($p = 0.0061$) in metastatic vs primary tumors, 52/79 (66%) vs 3/15 (20%). In 13 patients showing a good response of the primary tumor, 11 (85%) showed a good response in all their metastases. Conversely, of five patients with a poor histological response in the primary, two had a good response in metastases, two a poor response and there was one mixed response.

(4) What is the outcome, after intensive combined modality treatment with chemotherapy and surgery, in patients relapsing with metastases after previous adjuvant chemotherapy, and what are the important prognostic factors?

In Table 3, five studies[17–21] show the results of combined modality treatment with surgery and chemotherapy for patients relapsing after adjuvant treatment. Two studies[19,20] only report results in patients with lung metastases: these studies are heterogeneous in terms of prior therapy, subsequent treatments and outcomes reported. It is clear that some patients can be salvaged by aggressive treatment and the outlook may not be dissimilar to that reported for patients presenting with lung metastases (Table 2). In three of the four studies in which a prognostic factor analysis was performed, complete resection of metastases was independently correlated with improved survival.

In the multi-institutional study (MIOS), patients relapsing after surgery alone had a significantly longer interval to further disease progression ($p < 0.01$) and improved survival after relapse ($p = 0.01$) compared with those relapsing after treatment with immediate chemotherapy.[21]

(5) Can a biological agent (L-MTP-PE) prolong the time to relapse in patients with resected metastatic osteosarcoma?

Kleinerman and co-workers have published a series of studies on liposome-encapsulated muramyl tripeptide-phosphatidyl ethanolamine (L-MTP-PE) which has been shown to activate canine adherent mononuclear cells to become cytotoxic against canine osteosarcoma cells. This agent improved survival in a randomized trial when it was given as adjuvant therapy vs control in dogs undergoing amputation for osteosarcoma.[22] Preliminary *in vitro* studies[23] demonstrated that the tumoricidal properties of monocytes from 25 patients with osteosarcoma could be activated by L-MTP-PE to levels equal to, or greater than, those expressed by normal monocytes from control patients. Single-agent chemotherapy with DDP, DOX, HDMTX and cyclophosphamide did not interfere with this activation process and there was even a suggestion of enhanced activation potential following administration of DOX. In a subsequent study,[24] specific biological effects were shown in patients receiving infusions of L-MTP-PE, 2 mg $m^{-2}$ twice weekly $\times 12$, then weekly $\times 12$. These included rapid induction of circulating tumor necrosis factor (TNFα) and interleukin-6 (IL-6) as well as more prolonged elevations of C-reactive proteins and neopterin. This study went on to accrue 28 patients who had developed pulmonary metastases during adjuvant chemotherapy, or who presented with pul-

**Table 3.** *Systemic relapse—combined modality treatment/prognostic factors*

| Institution/Group (reference) | No. patients | No. prior chemotherapy | Sites | Treatment | Outcomes (from relapse) | Prognostic factors |
|---|---|---|---|---|---|---|
| Scandinavian Group[17] | 60 | 6 none<br>28 'low potency'<br>26 'intensive' | Lung 88% | Surgery 47%<br>Adequate chemotherapy 54% | 5 year OS 24% | Improved OS:<br>• solitary metastasis<br>• complete resection<br>• adequate chemotherapy |
| Gustav Roussey, Paris[18] | 42 | All | Lung 67% | Surgery 81%<br>Chemotherapy 57% | 3 year<br>DFS 27 %,<br>OS 36% | Improved OS:<br>• local/pulmonary recurrence<br>• complete resection |
| University of California, LA[19] | 36 | All | Lung only | Surgery + chemotherapy 100% | 5 year OS 23% | Improved OS:<br>• <4 nodules<br>• long disease-free interval<br>• unilateral lung metastases |
| Rizzoli, Bologna[20] | 25 | All | Lung only | High-dose IFOS 100%<br>Surgery 92% | 3 year DFS 44% | |
| MIOS[21] | 29<br>23 | None<br>Multi-agent | Lung 86%<br>Lung 78% | Surgery 71%<br>Chemotherapy 75% | 5-year DFS 51%<br>5-year DFS 24% | Improved OS:<br>• complete resection of all disease |

monary metastases that persisted despite chemotherapy. All patients were rendered free of visible disease by surgery before administration of L-MTP-PE. In five patients, a single tumor nodule recurred within 6 weeks of completing immunotherapy[25] and was resected. In three cases, histology of the tumor nodules showed peripheral fibrosis, an inflammatory cell infiltrate and neovascularity; the fourth showed early fibrosis and in the fifth the histology had changed from high to low grade. The clinical outcome was compared[26] between 12 patients receiving 12 weeks of therapy (group 1) and 16 patients receiving 24 weeks of therapy (group 2), as well as a similar group of historical controls (1980–1990) who received chemotherapy (group 3). There was a significant difference in time to relapse between groups 2 and 3 (9 vs 4.5 months). In nine patients, administration of L-MTP-PE in combination with IFOS 1.8 $g\ m^{-2}\ day^{-1} \times 5$ did not seem to increase toxicity and was well tolerated.[27] Immune activation was similar to that observed for L-MTP-PE alone and histological changes compatible with a chemotherapy effect (necrosis) and an immune effect (fibrosis with inflammatory cell infiltrate) were seen in three patients.

L-MTP-PE is currently (since November 1993) being evaluated in an intergroup trial (CCG 7921; POG 9351; INT 0133) in patients with non-metastatic osteosarcoma. Patients first receive induction chemotherapy with DOX/HDMTX with or without IFOS. After definitive surgery, they receive maintenance therapy with DOX/DDP/HDMTX and/or IFOS and are randomized between L-MTP-PE and control, given during and following maintenance chemotherapy, for a total of 36 weeks.

### Conclusions

(1) Long-term survival in osteosarcoma patients presenting with metastatic disease has been poor historically, especially for those with metastases in bone. Early results seem better for intensive multi-agent combinations incorporating IFOS, but these must be confirmed by long-term follow-up.

(2) In a non-randomized study, IFOS showed greater activity as a single agent in patients who had not received previous chemotherapy than in patients relapsing after prior chemotherapy. Thus, the 'therapeutic window' method may provide a better assessment of the efficacy of a new drug. CARBO showed low activity in previously untreated patients suggesting that it should not replace DDP in potentially curative regimens. Responses rarely occur in patients who have received three or more regimens of chemotherapy.

(3) When IFOS is incorporated in intensive multi-agent chemotherapy, histopathological good response rates are high (70–90%) in both primary tumors and pulmonary metastases. However, there may be technical variations in assessing cell kill in bone and lung.

(4) Based on 5-year survival figures, somewhere between one-quarter to one-half of patients with systemic relapse can be salvaged with intensive multi-modality therapy. The outlook seems better if metastatic disease can be completely resected.

(5) Studies have confirmed the immuno-stimulatory effects of L-MTP-PE and histopathologic studies suggest an inflammatory/fibrotic effect in lung metastases. A very small non randomized study has found extended DFS in patients receiving L-MTP-PE after resection of lung metastases, in comparison with historical controls.

### References

1 Bramwell VHC. The role of chemotherapy in osteogenic sarcoma. *Crit Rev Oncol/Hematol* 1995; 20:61–85.

2 Bramwell VHC. Chemotherapy of operable osteosarcoma. *Bailliere's Clinical Oncology* 1987; 1:175–203.

3 Rosen G, Marcove RC, Huvos AG, *et al.* Primary osteogenic sarcoma: eight year experience with adjuvant chemotherapy. *J Cancer Res Clin Oncol* 1983; 106:55–67.

4 Pacquement H, Blanchard C, Kalifa C, *et al.* Metastatic osteogenic sarcoma at diagnosis. Study of 73 cases treated by the French Society of Pediatric Oncology (SFOP) between 1980 and 1990. *Proc Am Soc Clin Oncol* 1995; 14:456.

5 Meyers PA, Heller G, Healey JH, *et al.* Osteogenic sarcoma with clinically detectable metastasis at initial presentation. *J Clin Oncol* 1993; 11:449–53.

6 Marina MN, Pratt CB, Rao, BN, *et al.* Improved prognosis of children with osteosarcoma metastatic to the lung(s) at the time of diagnosis. *Cancer* 1992; 70:2722–7.

7 Biagini R, Bacci G, Picci P, *et al.* Osteosarcoma of the extremities metastatic at presentation (OEMP). *Proc Am Soc Clin Oncol* 1992; 11:415.

8 Harris M, Gieser P, Link M, *et al.* Treatment of metastatic osteosarcoma at diagnosis. A Pediatric Oncology Group study. *Proc Am Soc Clin Oncol* 1995; 14:445.

9 Harris MB, Cantor AB, Goorin AM, *et al.* Treatment of osteosarcoma with ifosfamide: comparison of response in pediatric patients with recurrent disease versus patients previously untreated: a Pediatric Oncology Group study. *Medical and Pediatric Oncology* 1995; 24:87–92.

10 Bacci G, Mercuri M, Briccoli A, *et al.* Osteogenic sarcoma of the extremity with detectable lung metastases at presentation. Results of treatment of 23 patients with chemotherapy followed by simultaneous resection of primary and metastatic lesions. *Cancer* 1997; 79:245–54.

11 Ferguson W, Harris M, Link M, *et al.* Carboplatin in the treatment of newly-diagnosed metastatic or unresectable osteosarcoma: a Pediatric Oncology Group study. *Proc Am Soc Clin Oncol* 1996; 15:521.

12 Bieling P, Maerker I, Beron G, *et al.* Phase II study of carboplatin (CB) in pretreated disseminated osteosarcoma (OS). *Proc Am Soc Clin Oncol* 1992; 11:414.
13 Patel SR, Papadopoulos NE, Plager C, *et al.* Phase II study of paclitaxel in patients with previously treated osteosarcoma and its variants. *Cancer* 1996; 78:741–4.
14 Wierzbicki R, Ezzat A, Bazarbashi S, *et al.* Phase II trial of chronic daily VP-16 (Etoposide) administration in osteogenic sarcoma (OS). *Proc Am Soc Clin Oncol* 1993; 12:473.
15 Pappo A, Meyer W, Marina N, *et al.* Chemotherapy for recurrent osteosarcoma (OS): is it worth it? *Proc Am Soc Clin Oncol* 1992; 11:368.
16 Gentet JC, Brunat-Mentigny M, Kalifa C, *et al.* Ifosfamide (IFO) and etoposide (VP) in refractory or recurrent childhood osteosarcoma (OS). A phase II study of the French Society of Pediatric Oncology (SFOP). *Proc Am Soc Clin Oncol* 1995; 14:450.
17 Saeter G, Høie J, Stenwig AE, *et al.* Systemic relapse of patients with osteogenic sarcoma. Prognostic factors for long term survival. *Cancer* 1995; 75:1084–93.
18 Tabone M-D, Kalifa C, Rodary C, *et al.* Osteosarcoma recurrences in pediatric patients previously treated with intensive chemotherapy. *J Clin Oncol* 1994; 12:2614–20.
19 Ward WG, Mikaelian K, Dorey F, *et al.* Pulmonary metastases of stage IIB extremity osteosarcoma and subsequent pulmonary metastases. *J Clin Oncol* 1994; 12:1849–58.
20 Picci P, Bacci G, Ferrari S, *et al.* Salvage treatment with high-dose ifosfamide and surgery for osteosarcoma patients relapsed with lung metastases. Preliminary results. *Proc Am Soc Clin Oncol* 1996; 15:459.
21 Goorin AM, Shuster JJ, Baker A, *et al.* Changing pattern of pulmonary metastases with adjuvant chemotherapy in patients with osteosarcoma: results from the multi-institutional osteosarcoma study. *J Clin Oncol* 1991; 9:600–5.
22 McEwen EG, Kurzman ID, Rosenthal RC, *et al.* Therapy for osteosarcoma in dogs with intravenous injection of liposome-encapsulated muramyl tripeptide. *J Natl Cancer Inst* 1989; 81:935–8.
23 Kleinerman ES, Snyder JS, Jaffe N. Influence of chemotherapy administration on monocyte activation by liposomal muramyl tripeptide phosphatidylethanolamine in children with osteosarcoma. *J Clin Oncol* 1991; 9:259–67.
24 Kleinerman ES, Jia S-F, Griffin J, *et al.* Phase II study of liposomal muramyl tripeptide in osteosarcoma: the cytokine cascade and monocyte activation following administration. *J Clin Oncol* 1992; 10:1310–16.
25 Kleinerman ES, Raymond AK, Bucana CD, *et al.* Unique histological changes in lung metastases of osteosarcoma patients following therapy with liposomal muramyl tripeptide (CGP 19835A lipid). *Cancer Immunol Immunother* 1992; 34:211–20.
26 Kleinerman ES, Gano JB, Johnston DA, *et al.* Efficacy of liposomal muramyl tripeptide (CGP 19835A) in the treatment of relapsed osteosarcoma. *Am J Clin Oncol* 1995; 18:93–9.
27 Kleinerman ES. Biologic therapy for osteosarcoma using liposome-encapsulated muramyl tripeptide. *Hematology/Oncology Clinics of North America* 1995; 9:927–38.
